# Iron accumulation confers neurotoxicity to a vulnerable population of nigral neurons: implications for Parkinson’s disease

**DOI:** 10.1186/1750-1326-9-27

**Published:** 2014-07-10

**Authors:** Scott Ayton, Peng Lei, Paul A Adlard, Irene Volitakis, Robert A Cherny, Ashley I Bush, David I Finkelstein

**Affiliations:** 1 Florey Institute for Neuroscience and Mental Health, The University of Melbourne, Melbourne, Victoria, Australia

**Keywords:** Parkinson’s disease, Iron, Ceruloplasmin, Age

## Abstract

**Background:**

The substantia nigra (SN) midbrain nucleus is constitutively iron rich. Iron levels elevate further with age, and pathologically in Parkinson’s disease (PD). Iron accumulation in PD SN involves dysfunction of ceruloplasmin (CP), which normally promotes iron export. We previously showed that ceruloplasmin knockout (CP KO) mice exhibit Parkinsonian neurodegeneration (~30% nigral loss) by 6 months, which is prevented by iron chelation. Here, we explored whether known iron-stressors of the SN (1) aging and (2) MPTP, would exaggerate the lesion severity of CP KO mice.

**Findings:**

We show that while 5 month old CP KO mice exhibited nigral iron elevation and loss of SN neurons, surprisingly, aging CP KO mice to 14 months did not exacerbate iron elevation or SN neuronal loss. Unlike young mice, iron chelation therapy in CP KO mice between 9–14 months did not rescue neuronal loss. MPTP exaggerated iron elevation in young CP KO mice but did not increase cell death when compared to WTs.

**Conclusions:**

We conclude that there may exist a proportion of substantia nigra neurons that depend on CP for protection against iron neurotoxicity and could be protected by iron-based therapeutics. Death of the remaining neurons in Parkinson’s disease is likely caused by parallel disease mechanisms, which may call for additional therapeutic options.

## Findings

### Introduction

The substantia nigra pars compacta (SN) contains high iron and dopamine levels even in healthy individuals [1], which elevate further with age [2] and even more so in PD patients [3-5]. Iron elevation in PD SN occurs early in the disease [6], and correlates with motor disability [7]. Iron accumulation also features in animal models of PD such as MPTP intoxication, and likely contributes to cell loss since iron chelation is neuroprotective [4,5]. Importantly, iron chelation also improved motor symptoms of PD in a 12-month Phase II clinical trial [8] also suggesting that iron elevation confers neurotoxicity in the disease. While these data support a role for iron in contributing to the degenerative processes, it is unclear to what extent iron causes cell death in the disease, or if parallel disease mechanisms are more deleterious. Understanding the magnitude of iron-induced cell death in the disease will inform us if targeting iron pharmacologically will tangibly benefit PD patients.

Genetic disorders of brain-iron homeostasis can also present as Parkinsonism [9-11], demonstrating that iron accumulation can be a primary cause of PD-neurodegeneration. Aceruloplasminemia is a rare genetic disorder caused by loss of function mutations in ceruloplasmin (CP). CP is the major copper-binding protein of plasma, but it functions to maintain iron homeostasis. The protein coordinates 6 copper atoms which enables electron transport and reduction of oxygen to water, coupled with oxidation of iron. This chemistry is used to facilitate cellular iron export. Aceruloplasminemia is characterized biochemically by iron retention in brain and peripheral tissues, causing various pathologies such as diabetes, Parkinsonism, and dementia [12].

Loss of CP specific activity occurs in serum [13-16], CSF [17-19], and post-mortem SN tissue in PD [4]. Impaired CP activity, without the loss of CP abundance, might be caused by oxidative damage to the protein [19], leading to iron deposition in the disease. We and others have previously modelled loss of CP activity using CP knockout (KO) mice [4,20]; these mice develop iron-dependent Parkinsonism before 6 months of age [4]. Here we explore if stressing iron homeostasis further in CP KO mice with the PD toxin, MPTP, or by aging, would exaggerate the PD phenotype, in order to understand the contribution of iron elevation to progressive SN neurodegeneration.

## Methods

### Ethics statement

All animal experiments were approved by the Howard Florey Animal Ethics Committee and were conducted in accordance with state law and the Australian Code of Practice for the Care and Use of Animals for Scientific Purposes as described by the National Health and Medical Research Council of Australia.

### Mice

At the conclusion of each experiment, mice were transcardially perfused with ice-cold PBS under deep anaesthesia (sodium pentobarbitone, 100 mg/kg). When blood was harvested, it was collected in lithium heprin tubes. Tissues were microdissected and analysed by inductively coupled plasma mass spectrometry (ICP-MS; Additional file 1: Supplementary Methods).

### Ageing study

CP KO mice and background C57/Bl6 mice (WT) were euthanized at either 5 or 14 months. Microdissected brain was either sectioned for stereology or homogenized in PBS and analysed for metals ICP-MS. Peripheral organs were also homogenized in PBS for ICP-MS analysis.

### Chronic chelation trial

9-month-old CP KO mice were administered either clioquinol (Cq) or deferiprone (DFP) iron chelators for five months. DFP (Sigma Medical) was administered in drinking water (≈100 mg/kg/day; ad libitum) as previously described [4]. Mice treated with Cq were fed a diet of rodent chow mixed with 0.25 g/kg (≈30 mg/kg/day; ad libitum) of Cq (Specialty Feeds, Western Australia), as previously described [5]. Mice were euthanized at 14 months of age and midbrain was removed, sectioned and SN neurons were counted using stereology.

### MPTP trial

5-month-old WT and CP KO mice were administered 50 mg/kg MPTP (4 × 12.5 mg/kg, 2 hours apart, I.P.). 24 hours later, mice of both genotypes were administered either DFP (as described above) or purified ceruloplasmin (CP) protein. CP was administered (5 mg/kg in saline, I.P.; Vital products) twice weekly (day 2, 4, 8, 11, 15 & 18). After 21 days, mice were euthanized. Microdissected brain was either sectioned for stereology or homogenized in PBS and analysed for metals ICP-MS. Peripheral organs were also homogenized in PBS for ICP-MS analysis.

## Results

**Figure 1:**
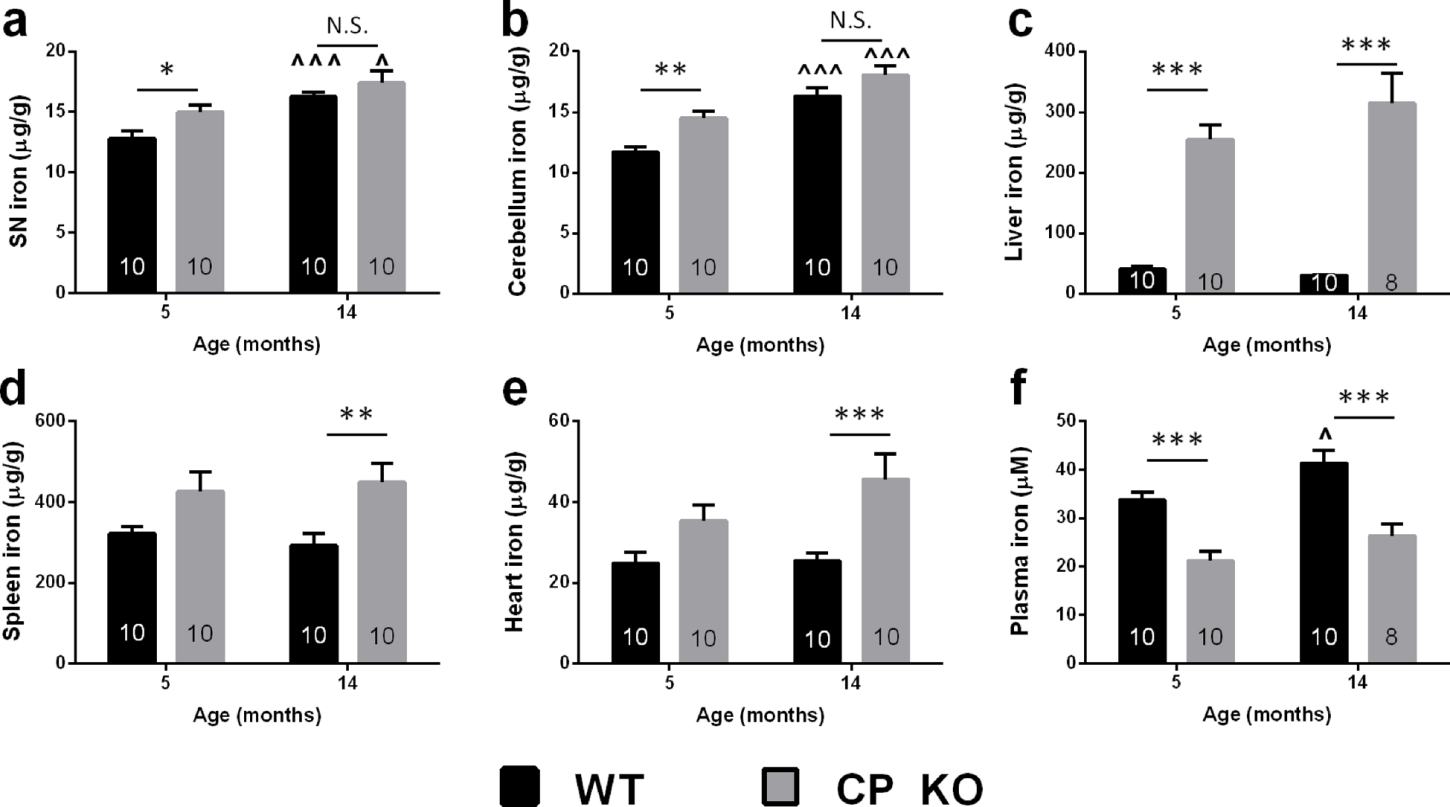
**Iron levels in CP KO mice tissue.** WT and CP KO mice were aged to 5 and 14 months. Iron was measured in homogenized **(a)** substantia ngira **(b)** cerebellum **(c)** liver **(d)** spleen and **(e)** heart by ICP-MS and corrected for tissue weight. **(f)** Plasma extract from blood was measured for iron by ICP-MS. Data are mean ± std error. ‘n’ is represented in graph columns. *p < 0.05, **p < 0.01, ***p < 0.001 as indicated. ^p < 0.05, ^^^p < 0.001 relative to 5-month-old of the same genotype.

Iron accumulation occurs in the brain with age [21]. First, we explored whether this process was exaggerated in CP KO mice, which have impaired iron export (Figure 1). Iron was elevated in nigra (~20%, *P* = 0.034; Figure 1a) and cerebellum (CB; ~40%, *P* = 0.002; Figure 1b) of 5-month-old CP KO mice compared to WT mice, similar to findings we reported for mice aged 6 months [4]. Aging mice to 14 months caused iron to accumulate in both brain regions of WT mice (SN: *P* < 0.001; CB: *P* < 0.001; Figure 1a,b) so that CP KO mice at 14 months of age no longer had significantly elevated iron in these regions compared to similarly aged WTs. The stress of aging, therefore, did not exaggerate iron accumulation in CP KO mouse brain compared to WTs.

Iron accumulation in CP KO mice was most pronounced in the liver, which is the major site of CP production in the body. CP KO liver exhibited marked iron elevation at both 5 (~500%, *P* < 0.001) and 14 months (~900%, *P* < 0.001; Figure 1c). A trend toward iron elevation was observed in the spleen and heart of CP KO mice at 5 months of age, which became significant at 14 months (Spleen:~50%, *P* = 0.005; Heart:~80%, *P* = 0.001; Figure 1d,e). Iron accumulation in peripheral tissues of the CP KO mouse was, in contrast to brain tissue, more apparent with age. Opposite to brain and peripheral tissues, plasma iron was decreased in CP KO mice (~35% at both 5 & 14 months, *P* < 0.001; Figure 1f). This may be because iron export from tissues to plasma is restricted due to the lack of CP, resulting in accumulation within tissues, and a loss of iron in plasma. Elevated iron in brain, and lower iron in plasma, is consistent with a Mendelian Randomization study which showed SNPs that increased serum iron levels decreased the risk for PD [4,22].

**Figure 2:**
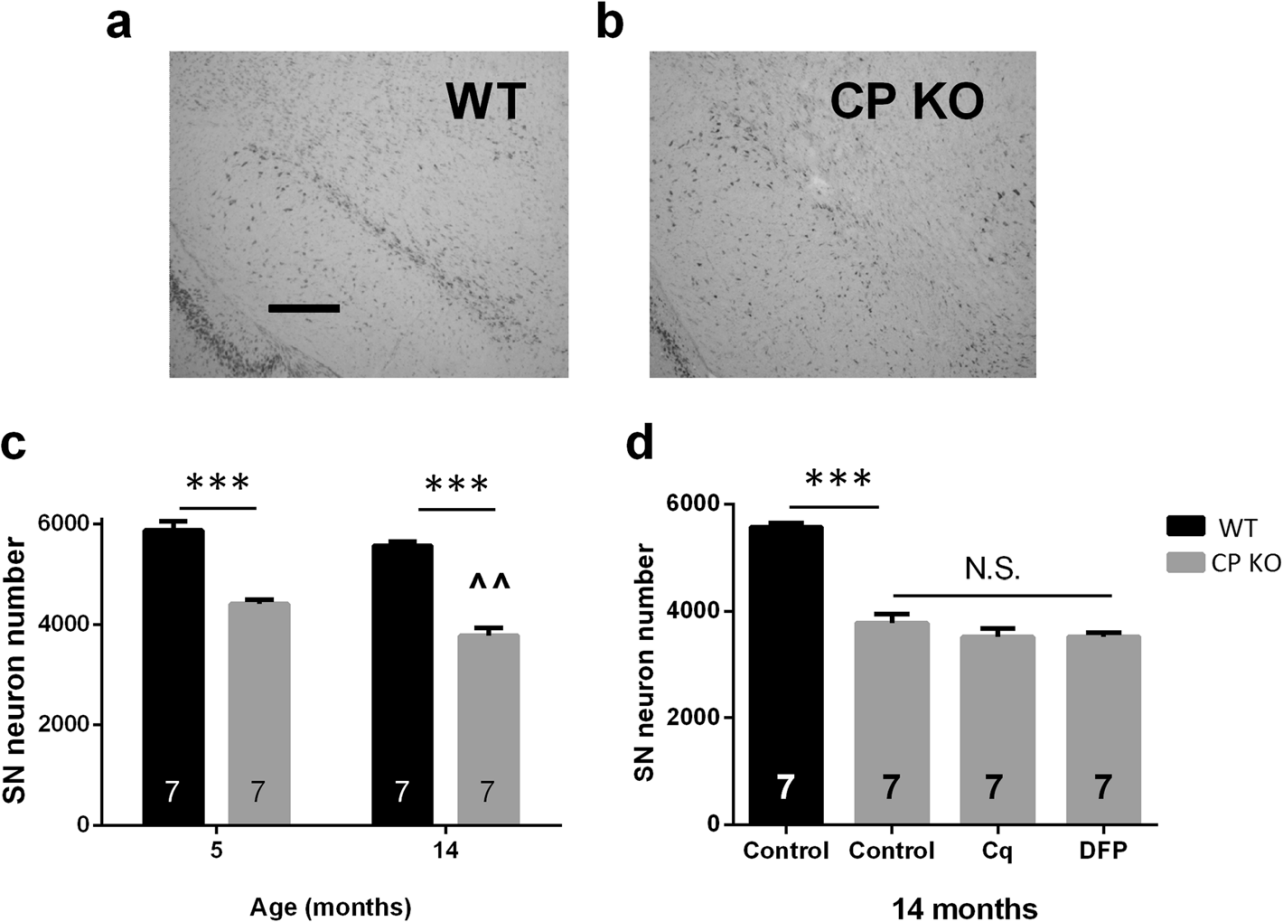
**Nigral neurons in CP KO mice treated chronically with iron chelators. (a-c)** WT and CP KO mice were aged to 5 and 14 months. Mice were euthanized and brains were harvested and midbrains sectioned **(a)** representative WT nigra **(b)** representative CP KO nigra **(c)** Nissl stained neurons were counted using stereology. **(d)** WT and CP KO mice were aged to 14 months. A sub-set of CP KO mice were treated with Cq and DFP daily from 9 months of age. Mice were euthanized and brains were harvested and midbrains sectioned. Data are mean ± std error. ‘n’ is represented in graph columns. ***p < 0.001 as indicated. ^^p < 0.01 relative to 5-month-old of the same genotype.

SN neuronal loss in 6 month old CP KO mice involves iron elevation, since iron chelation ameliorates this lesion [4,20]. We explored whether aging CP KO mice would cause more pronounced neurodegeneration. 5-month-old CP KO mice exhibited SN neurodegeneration (-25%, *P* < 0.001; Figure 2a) similar to our previous report [4] and aging caused further SN neuronal loss in CP KO mice (-32%, *P* < 0.001; Figure 2a). Only a further 7% of SN neurons deteriorated in the 9 months after the initial time point, demonstrating that the velocity of degeneration was clearly slowed at 14 months of age. This coincided with proportional disability on the Rotarod apparatus (Additional file 1: Figure S2a) and also increases in nigral iron levels between genotypes of the same age (Figure 1a). There was no difference between genotypes in cognitive performance in the Y-maze at either age (Additional file 1: Figure S2b).

To examine further the relationship between age and SN neuronal loss in CP KO mice we explored if iron chelation therapy would ameliorate neuronal loss in older CP KO mice, as it did in mice treated between 3 and 6 months of age [4]. CP KO mice were treated with iron chelators, deferiprone (DFP; 100 mg/kg/day) or clioquinol (Cq; 30 mg/kg/day), from 9 months of age until the experiment was terminated when mice were aged 14 months. Both Cq and DFP failed to rescue SN neuronal cell number over the treatment period (Figure 2b).

**Figure 3:**
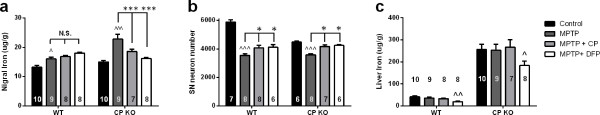
**MPTP-induced SN neuron loss in WT and CP KO mice is ameliorated with CP or DFP.** 5-month-old WT and CP KO mice were administered MPTP (50 mg/kg, I.P.) and euthanized 21 days after. A sub-set of WT and CP KO mice were treated with dosing regimens of CP or DFP during the experimental period. **(a)** RHS SN was microdissected and iron was measured by ICP-MS. **(b)** LHS midbrain was sectioned and Nissl stained neurons were counted by stereology. **(c)** Liver was harvested and measured for iron by ICP-MS. Data are mean ± std error. ‘n’ is represented in graph columns. *p < 0.05, ***p < 0.001 as indicated. ^p < 0.05, ^^p < 0.01, ^^^p < 0.001 relative to control of the same genotype.

Since the stress of aging did not exaggerate the velocity of SN neurodegeneration in CP KO mice, we hypothesized that a certain proportion of SN neurons were vulnerable to iron accumulation, and that these neurons were lost early in CP KO mice. To test this, we administered the PD toxin, MPTP, to WT and CP KO mice, which causes loss of CP expression and consequent iron elevation [4,23]. If the surviving SN neurons in CP KO mice at 5 months of age were comparatively resistant to iron toxicity, MPTP should not exaggerate nigral neuronal loss. MPTP caused nigral iron accumulation in WT mice (~30%, *P* < 0.001; Figure 3a), which was elevated further in CP KO mice (~60%, *P* < 0.001; Figure 3a). MPTP caused SN neuronal loss in both WT (-35%; *P* <0.001) and CP KO (-20%; *P* <0.001). Despite the increased iron burden in the MPTP-affected CP KO mice, neuronal loss in CP KO mice did not exceed that of WT mice (~3500 remaining neurons for both genotypes; *P* >0.05; Figure 3b).

We also investigated if targeting iron ameliorated MPTP-mediated neuronal death in both genotypes. We used two methods of targeting iron, CP supplementation therapy, which promotes cellular iron export, and DFP, which binds free iron. CP [4] and DFP [24] have been shown previously to rescue MPTP neurotoxicity and, as expected, lessened MPTP-induced neuronal loss in WT and CP KO mice (Recovered to ~4100 neurons in each case; Figure 3b). Rescue of iron elevation was evident in CP KO mice for both CP and DFP (Figure 3a), but the bulk iron levels in WT MPTP nigra were unchanged by either treatment.To test for drug specificity, we measured liver iron content in the same mice. MPTP did not change iron levels in either WT or CP KO mice (Figure 3c). CP treatment did not lower liver iron levels in either genotype, despite very high levels of iron in CP KO liver. DFP treatment, however, lowered iron in both WT and CP KO liver.

## Discussion

We hypothesized that iron-mediated neurodegeneration caused by loss of CP would be exaggerated with the stress of ageing, or with the MPTP toxin, which both induce iron accumulation. However, we show that aging slows the rate of SN iron accumulation (Figure 1a) and consequent neurodegeneration (Figure 2a) in CP KO mice, and MPTP fails to induce more pronounced neurodegeneration in CP KO mice (Figure 3b), despite exaggerating iron elevation in their nigra (Figure 3a). We propose that a proportion of SN neurons are more vulnerable to loss of CP, and associated iron neurotoxicity. This has implications for understanding iron-induced neurodegeneration in PD, and for pharmacologically targeting iron in the disease.

It is unknown why iron elevates in the SN during the aging process, but this could increase the susceptibility for PD by causing oxidative stress (which is a feature of the disease [25]) or possibly by Ferroptosis [26]. We show that a proportion of SN neurons are vulnerable to iron toxicity caused by genetic lesion (CP KO; Figure 2a), or by a PD toxin (MPTP; Figure 2b). The vulnerability of cells in this nucleus possibly explains why rare genetic disorders of iron dyshomeostasis that cause brain-wide iron elevation often present with Parkinsonism [9-11]. Toxicity of this proportion of neurons is responsive to therapies that target iron [4] (Figure 3a,b), which supports the use of iron-based therapies for PD.

Stressing the nigra of CP KO mice that already exhibit iron-dependent neurodegeneration (5 month old) with (1) iron accumulation caused by normal aging (Figure 2), or (2) MPTP (Figure 3), did not exaggerate the loss of SN neurons, suggesting that the surviving neurons are resistant to iron-induced neurodegeneration. Possibly, the neurons most vulnerable to MPTP-induced iron elevation depend on CP to protect them, and that MPTP induces a loss of CP [4] that causes these neurons to accumulate iron and die. The remaining neurons of the SN do not rely as much on CP for protection possibly because they have added defences against the consequences of iron elevation.

In the MPTP model, two different pharmacological interventions targeting iron (CP and DFP) were neuroprotective to CP KO and WT mice (Figure 3b), however we observed that treatments only decreased bulk iron levels in CP KO, not WT nigra (Figure 3a). Both CP and DFP lower the neuronal labile iron pool, thus preventing iron- redox interactions, so it is not necessary for bulk iron levels to be reduced in DFP-treated animals for DFP to confer neuroprotection by iron chelation. We previously showed that CP supplementation lowered nigral iron levels in MPTP-administered mice [4]. The CP dosing regimen in the present work was different to our previous study [4], concluding three days prior to termination of the experiment, which may have allowed time for iron to accumulate.

Our current findings indicate that there are some SN neurons that depend on CP expression to protect them from iron induced death, and there other neurons that have an alternative protection mechanism that does not rely on CP. Therefore, there is an opportunity to treat PD by pharmacologically removing iron that causes cell death in vulnerable neurons. This is important because iron-mediated neurodegeneration can be targeted by established therapies, like iron chelation, or biological alternatives such as ceruloplasmin therapy. Indeed, DFP is the first drug to show slowing of the disease progression in a clinical trial [8], which highlights the promise of this approach. Since iron elevation did not exacerbate neuronal cell death beyond ~30% in our study it is likely that parallel mechanisms contribute to the degenerative process in PD. So while there is opportunity to treat some cells of the SN with iron chelators, there likely exists a proportion of dying neurons in the disease that are intractable to iron-based therapies.

## Abbreviations

CP: Ceruloplasmin; Cq: Clioquinol; DFP: Deferiprone; KO: Knockout; PD: Parkinson’s disease; SN: Substantia nigra; ICP-MS: Inductively coupled plasma mass spectrometry; MPTP: 1-methyl-4-phenyl-1,2,3,6-tetrahydropyridine.

## Competing interests

Drs. Adlard, Cherny and Finkelstein are shareholders in and paid scientific consultants for Prana Biotechnology Pty Ltd. Dr. Bush is a shareholder in Prana Biotechnology Pty Ltd., Eucalyptus Pty Ltd., Mesoblast Pty Ltd. and a paid consultant for Collaborative Medicinal Developments LLC. Drs Bush, Finkelstein and Ayton have a patent relating to this work.

## Authors’ contributions

Scientific concept: SA, PL, DIF, AIB. Experimental design: SA, PL, PAA, RAC, IV, DIF, AIB. ICP-MS: IV, RAC. Stereology: SA, PL. Drug treatments: PAA. Wrote the manuscript: SA, PL, AIB, DIF. Edited the manuscript, SA, PL, PAA, RAC, IV, DIF, AIB. All authors read and approved the final manuscript.

## Supplementary Material

Additional file 1 **Supplementary Results.** Supplementary Methods. Click here for file
