# Supplementary material for: Iron accumulation confers neurotoxicity to a vulnerable population of nigral neurons: implications for Parkinson’s disease
Source: Mol Neurodegener. 2014 Jul 10;9:27. doi: 10.1186/1750-1326-9-27 (PMC4114139; doi:10.1186/1750-1326-9-27)
Supplement: Additional file 1 — Supplementary Results. Supplementary Methods. [file 1750-1326-9-27-S1.pdf]

Iron accumulation confers neurotoxicity to a vulnerable population of nigral neurons: implications for Parkinson's disease

Scott Ayton, Peng Lei, Paul A. Adlard, Irene Volitakis, Robert A. Cherny, Ashley I. Bush and David I. Finkelstein*

Supplementary Results

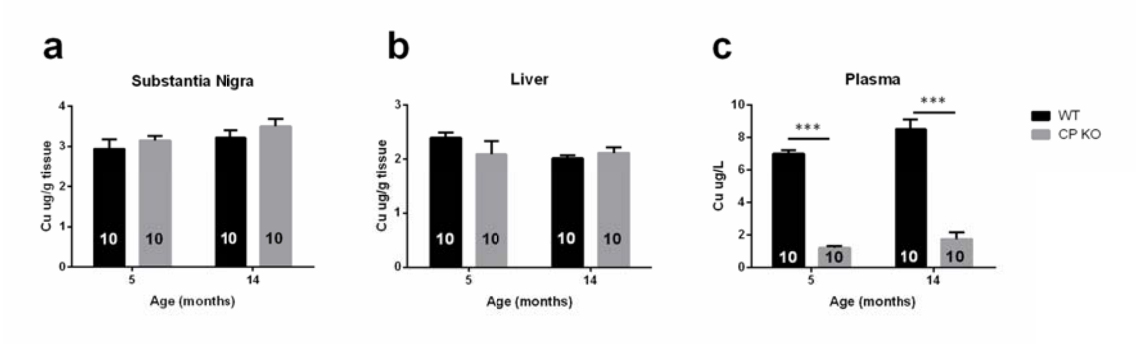

Supplementary Figure 1. Copper levels in CP KO mice tissue. WT and CP KO mice were aged to 5 and 14 months. Copper was measured in homogenized **(a)** substantia nigra and **(b)** liver by ICP-MS and corrected for tissue weight. **(c)** Plasma extract from blood was measured for copper with ICP-MS. Data are mean ± std error. 'n' is represented in graph columns. ***$p<0.001$.

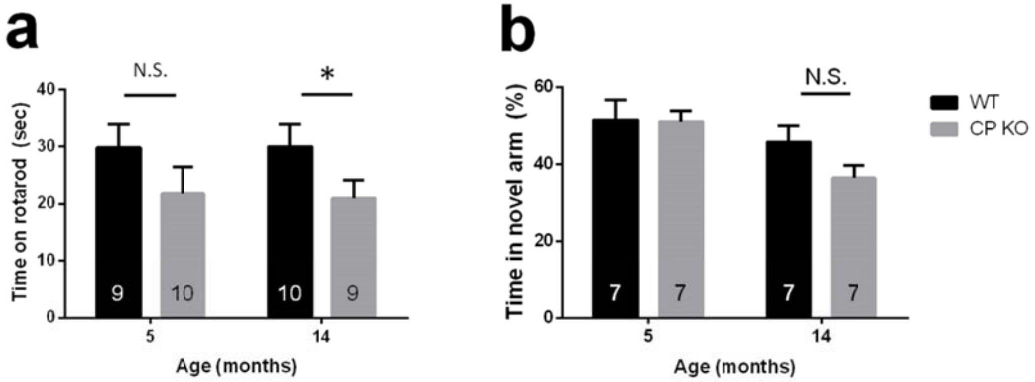

Supplementary figure 2. Analysis of motor and cognitive performance in young and old CP KO mice. WT and CP KO mice were aged to either 5 or 14 months. **(a)** Mice were tested for motor ability with the Rotarod apparatus. Decreased time spent on the Rotarod is consistent with motor disability. **(b)** Spatial memory was analysed in mice using the Y-maze.

Decreased time spent exploring the novel arm of the maze is consistent with poorer cognition. Data are mean ± std error. 'n' is represented in graph columns. *$p<0.05$.

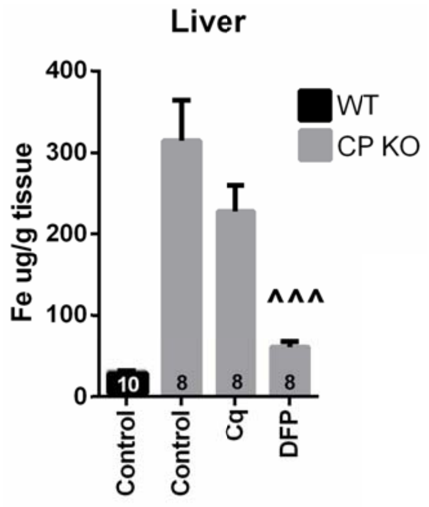

Supplementary figure 3. WT and CP KO mice were aged to 5 and 14 months. Mice were euthanized and liver harvested. Metals iron content in liver was measured by ICP-MS and corrected for weight of tissue. Data are mean ± std error. 'n' is represented in graph columns. ^^^$p<0.001$ relative to control of the same genotype.

<u>Supplementary Methods</u>

<u>Stereology:</u>

Nissl-stained substantia nigra pars compactor (SN) neurons were counted by blinded and unbiased stereology using our previously described methods[8]. Briefly, the midbrain was micro-dissected and then cut (30 µm) in 1:3 series with a cryostat, then stained with neutral red (Grale Scientific). The neurons in the SN were counted according to the optical fractionator rules, and the neurons in the SN were distinguished by the anatomical location, orientation, presence of nucleolus and cell density. Neuron numbers were estimated using an unbiased counting frame of x=35 µm, y = 45 µm (1575 μm^2) at regular intervals on a sampling grid of 140 x 140 (x= 140 µm, y= 140 µm), and a stereological program (Stereoinvestigator, Microbrightfield, USA) viewed with a 63 x 1.3 N.A. oil objective (DMLB Leica Microscope).

ICP-MS:

Brain and organ samples were weighed and homogenized. An aliquot was freeze-dried, and then resuspended in nitric acid (65% Suprapur, Merck) overnight. The samples were then heated for 20 min at 90 ˚C, and equivalent volume of hydrogen peroxide (30%, Merck) was added for a further 15 min incubation at 70 ˚C. Iron and copper in the digested sample was measured using an Agilent 7700 series ICP-MS instrument under routine multi-element operating conditions using a Helium Reaction Gas Cell. The instrument was calibrated using 0, 5, 10 , 50, 100 and 500 ppb of certified multi- element ICP-MS standard calibration solutions (ICP-MS-CAL2-1, ICP-MS-CAL-3 and ICP-MS-CAL-4, Accustandard) for a range of elements. We used a certified internal standard solution containing 200 ppb of Yttrium (Y89) as an internal control (ICP-MS-IS-MIX1-1, Accustandard). Each sample was measured in triplicate, and the concentrations determined from the standard curve were normalized to wet tissue weight.

Rotarod

Mice were placed on a rotating rod (Panlab, Barcelona, Spain), spinning at 4 RPM (Lane width, 50mm, rod diameter 30mm). Once stabilised, mice were subjected to an incrementally increasing speed of 1 RPM per 8 seconds. Each animal underwent 3 trials. The length of time that the mice managed to remain on the rod, and the speed at which they fell off the apparatus, were recorded. The average of the three trials was used for further analysis.

Y-maze

The Y maze test utilizes a Y-shaped grey-painted timber with arms 29.5cm long × 7.5cm wide × 15.5 cm. All mice were subjected to a 2-trial Y-maze test separated by a 1-h inter-trial interval to assess spatial memory, with all testing performed during the light phase of the circadian cycle. The 3 identical arms were randomly designated start arm, novel arm, and other arm. Visual cues were placed on the walls of the maze. The first trial (training) was for 10 min, and the mice were allowed to explore only 2 arms (starting arm and other arm). For the second trial (retention) mice were placed back in the maze in the same starting arm, and

allowed to explore for 5 min with free access to all 3 arms. Behaviours were recorded on video during a 1 min trial and Ethovision video-tracking system (Noldus, Netherlands) was used for analysis. The amount of time spent in the novel arm is indicative of spatial memory. Where mice exhibit spatial memory impairment, the amount of time spent in the novel arm should approximate the amount of time spent in each of the other two arms (therefore, 33% in each arm). Data are expressed as the percentage of duration mice spent in the novel during the 1-min second trial.

Statistics:

All statistical procedures were performed with SPSS version 14.0 software (Lead Technologies). Two-factor ANOVA was performed in each case except for Figure 2b, where one-factor ANOVA was employed. Dunnett’s test was used for each post hoc test in the study. Two-tailed comparisons are used for each analysis.
